# PHACE (Posterior Fossa Brain Malformations, Hemangiomas, Arterial Anomalies, Cardiac Defects, and Eye Abnormalities) Syndrome in a Newborn With Bilateral Ocular Hemangiomas: The Importance of Early Recognition and Multidisciplinary Management

**DOI:** 10.7759/cureus.106213

**Published:** 2026-03-31

**Authors:** Claire M Overholt, Michael L Middleton, Michele N Lossius, Juan C Gonzalez, Jr.

**Affiliations:** 1 College of Medicine, University of Florida, Gainesville, USA; 2 Pediatrics, University of Florida College of Medicine, Gainesville, USA; 3 Emergency Medicine, University of Florida College of Medicine, Gainesville, USA

**Keywords:** congenital vascular anomaly, infantile hemangioma, neurocutaneous syndrome, phace, phace syndrome

## Abstract

PHACE (posterior fossa brain malformations, hemangiomas, arterial anomalies, cardiac defects, and eye abnormalities) syndrome is a rare neurocutaneous disorder characterized by large segmental infantile hemangiomas and associated congenital anomalies involving the brain, heart, eyes, and arterial vasculature. We present a case of a female infant with vascular lesions on the left periocular face, a midline chest and abdominal defect, and imaging findings of Dandy-Walker malformation, bilateral orbital hemangiomas, and cerebrovascular anomalies. She was ultimately diagnosed with PHACE syndrome based on clinical criteria. This case highlights the importance of early recognition of large facial hemangiomas as potential indicators of PHACE syndrome and the need for timely multidisciplinary evaluation to reduce the risk of serious complications such as stroke, seizure, visual and cognitive impairment, or critical aortic arch stenosis.

## Introduction

PHACE syndrome (posterior fossa brain malformations, hemangiomas, arterial anomalies, cardiac defects, and eye abnormalities) is a rare, neurocutaneous disorder characterized by large segmental infantile hemangiomas and associated congenital anomalies [1]. Diagnosis is based on consensus-derived criteria requiring the presence of a large segmental facial infantile hemangioma plus at least one major or two minor extracutaneous anomalies across the defining feature categories [2]. Early recognition and multidisciplinary evaluation are critical to preventing complications. We present a case that emphasizes the importance of prompt diagnosis and proper management of PHACE syndrome in a newborn with complex cerebrovascular, cardiac, and cutaneous findings.

PHACE syndrome affects fewer than one per million children in the general population, though prevalence rises to 2%-3% among infants presenting with infantile hemangiomas and up to 20%-31% when hemangiomas are large, segmental, and facial [3,4]. We present this case because of its unusually broad multisystem involvement at initial presentation, including bilateral orbital hemangiomas, Dandy-Walker malformation, cerebrovascular anomalies, and a ventral midline defect. This underscores the diagnostic complexity and the critical importance of early specialist referral.

## Case presentation

A female infant was born at 41 weeks of gestation via spontaneous vaginal delivery to a G2P1 mother. There were no complications of pregnancy or delivery. The infant was born with vascular lesions on the left periocular face and a linear midline skin defect from the umbilicus to the lower sternum, initially interpreted as a healing laceration during the birthing process. A head ultrasound shortly after birth revealed a mega cisterna magna.

At one month of life, the patient had developed bilateral periocular swelling, worsening facial lesions, and new left eye lid drainage. She presented to a local emergency department, where she was treated with oral erythromycin and subsequently admitted for continuation of symptoms despite oral antibiotics. Further head imaging with magnetic resonance imaging (MRI) was completed, which characterized the mega cisterna magna seen on initial ultrasound as a Dandy-Walker malformation, with findings of a hypoplastic vermis and cystic enlargement of the fourth ventricle. Dermatology noted a suspected port-wine stain on the face and an ulcerated abdominal/chest lesion, which was treated with mupirocin ointment.

**Figure 1:**
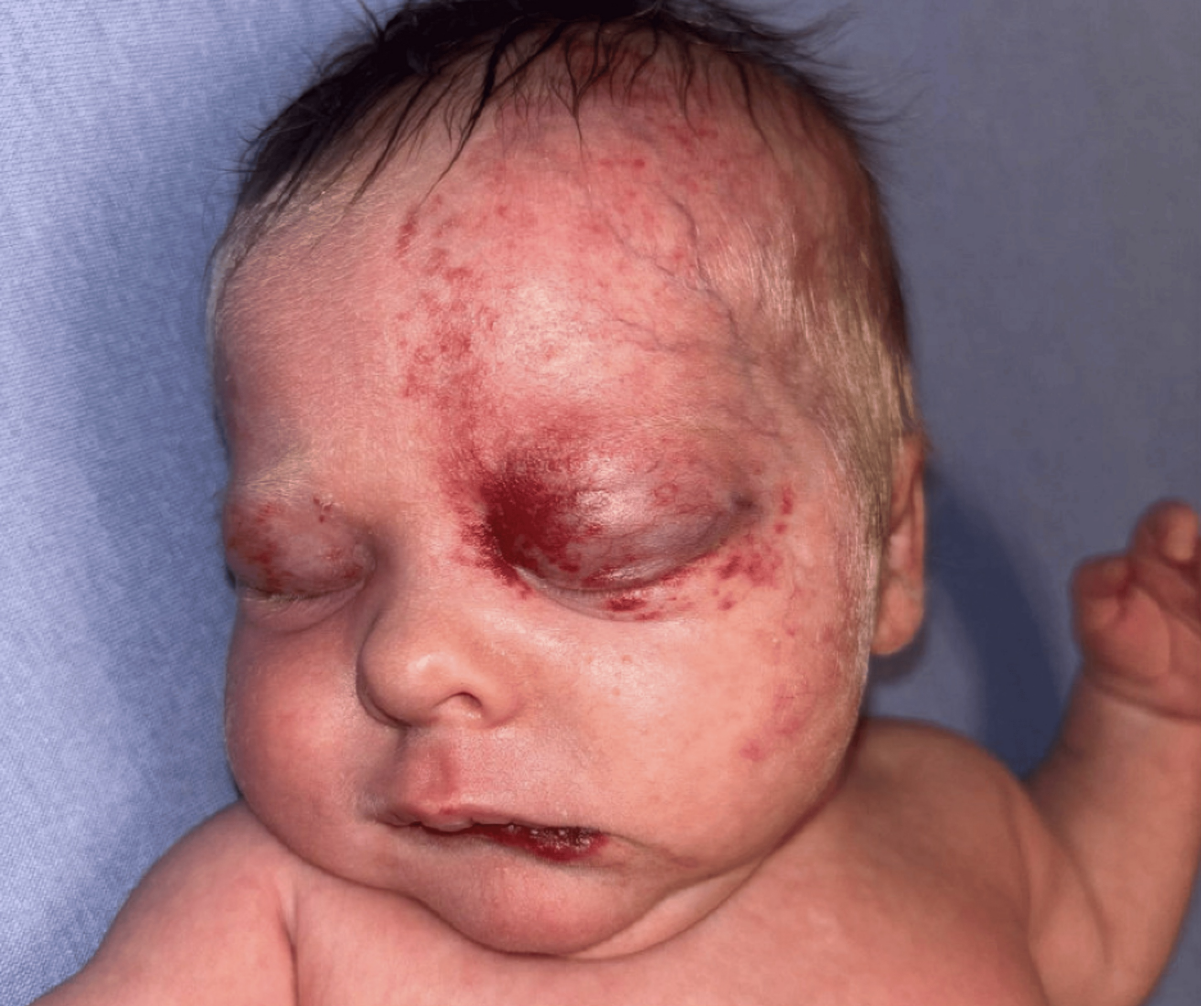
Clinical presentation showing vascular lesion and periorbital swelling during presentation to our emergency department, demonstrating the extent of left-sided facial hemangioma involvement and bilateral periocular swelling

Four days after discharge from their first admission at the local ED, the patient presented to our pediatric emergency department with fever, vomiting, diarrhea, poor oral intake, and worsening periocular swelling (Figure 1).

**Figure 2:**
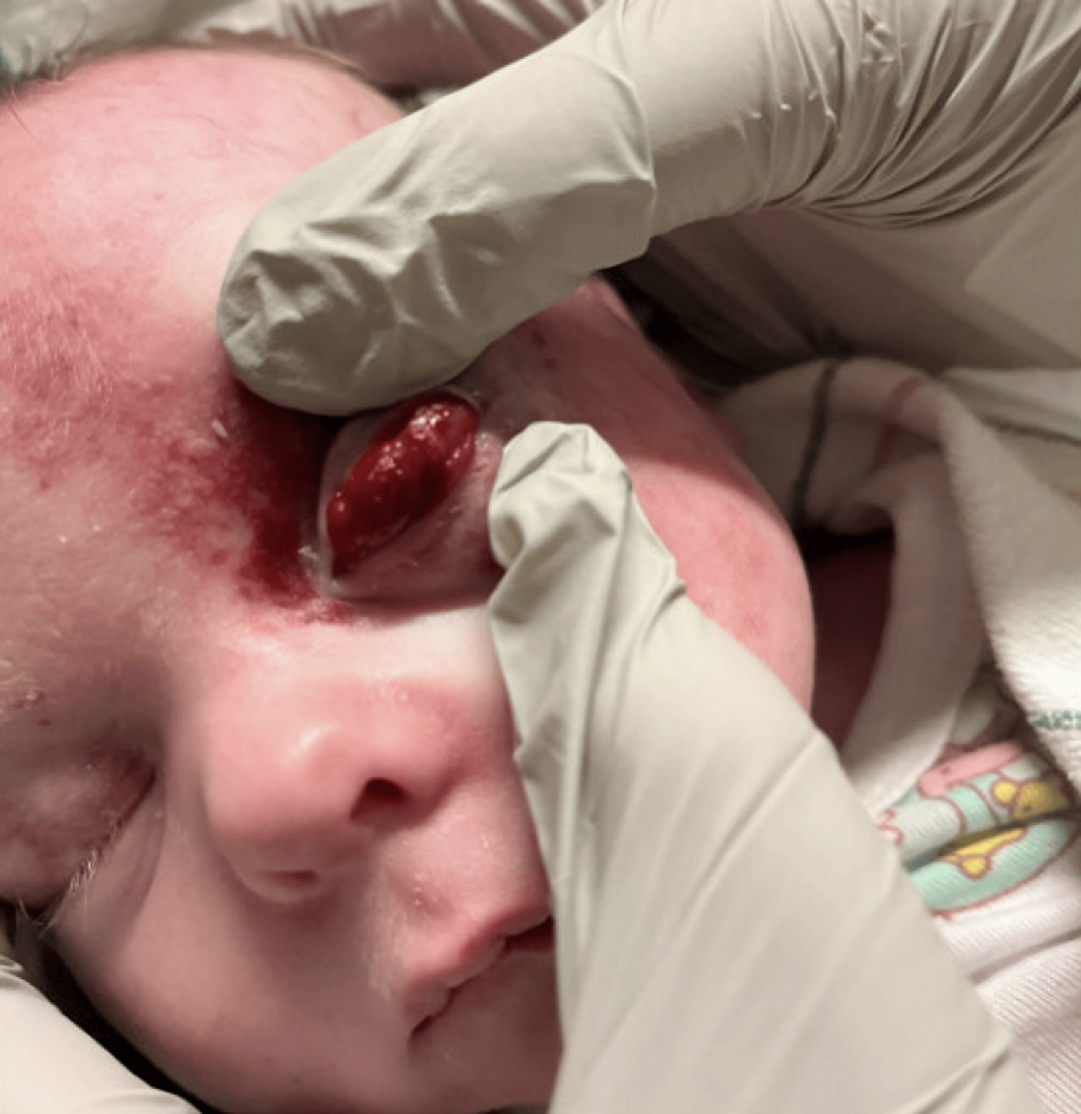
Clinical presentation highlighting capillary hemangioma on the left eyelid, a hallmark cutaneous feature of PHACE syndrome with potential to cause amblyopia through visual axis obstruction PHACE: Posterior fossa brain malformations, hemangiomas, arterial anomalies, cardiac defects, and eye abnormalities.

**Figure 3:**
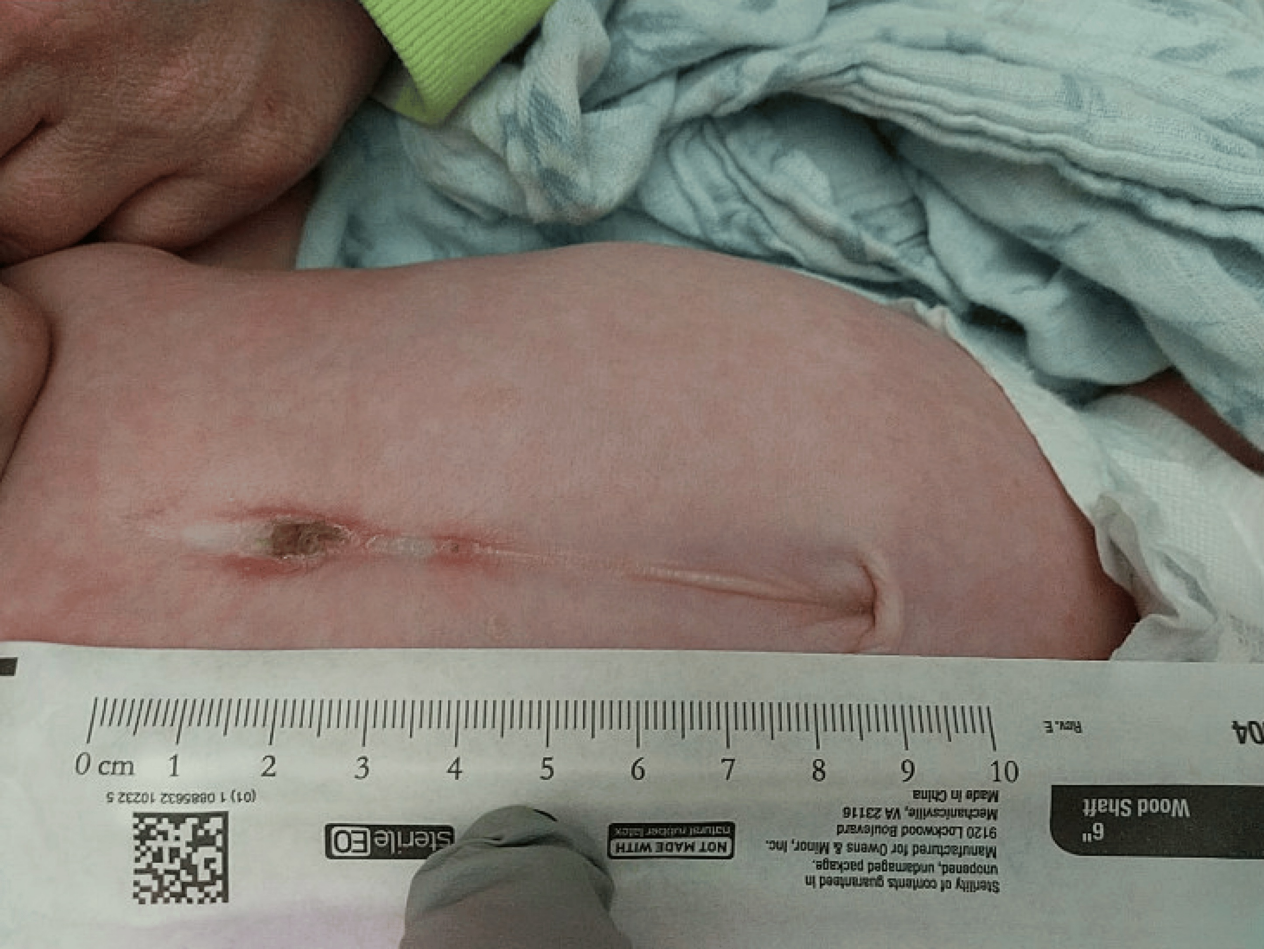
Clinical presentation showing supraumbilical raphe, a ventral midline defect and major diagnostic criterion for PHACE syndrome PHACE: Posterior fossa brain malformations, hemangiomas, arterial anomalies, cardiac defects, and eye abnormalities.

Physical examination revealed a prominent capillary hemangioma of the left eyelid and milder right-sided involvement, as well as a supraumbilical raphe (Figures 2, 3). Initial labs noted an incidental urinary tract infection (UTI). After review of outside imaging, given concern for possible PHACE syndrome, the patient was admitted to the general pediatric inpatient team for further imaging studies and inpatient consultations by ophthalmology, dermatology, and genetics.

While admitted, the patient underwent further imaging studies, which confirmed Dandy-Walker malformation, white matter deficiency, periventricular neuronal heterotopia, and the left orbital hemangioma. An ultrasound of the chest lesion revealed a 6 mm superficial soft tissue defect without vascular involvement, consistent with the supraumbilical raphe noted on examination. Ophthalmology evaluated the patient and recommended timolol drops, which showed immediate improvement in the swelling of the orbital hemangiomas. She was transitioned to oral antibiotics for her UTI and was seen by genetics after discharge.

Pediatric genetics evaluated the patient and diagnosed PHACE syndrome based on clinical criteria [2]. Following an outpatient genetics evaluation, the patient was readmitted to our institution for cerebrovascular imaging and inpatient monitoring before initiating propranolol. Magnetic resonance angiography (MRA) showed high-flow vascular malformations in the facial, temporal, and suboccipital regions, bilateral orbital hemangiomas (left > right), tortuous extracranial carotid arteries, and dysplastic segments of the left middle cerebral artery (MCA) and posterior cerebral artery (PCA). These cerebrovascular findings, particularly the dysplastic arterial segments, placed the patient in a high-risk category for arterial ischemic stroke, directly informing the decision to initiate antiplatelet therapy. Echocardiogram demonstrated a moderate atrial septal defect and a small patent ductus arteriosus. Renal ultrasound showed mild bilateral pelviectasis without hydronephrosis. The patient was started on low-dose aspirin (4 mg/kg/day) for elevated stroke risk, along with propranolol (0.5 mg/kg/day) and prednisolone (1 mg/kg/day) while admitted to the pediatric intensive care unit (PICU). All three medications were continued upon discharge. Prednisolone was planned for a short course and discontinued after two weeks, while propranolol was gradually uptitrated to a maintenance dose of 3 mg/kg/day over the following weeks.

**Figure 4:**
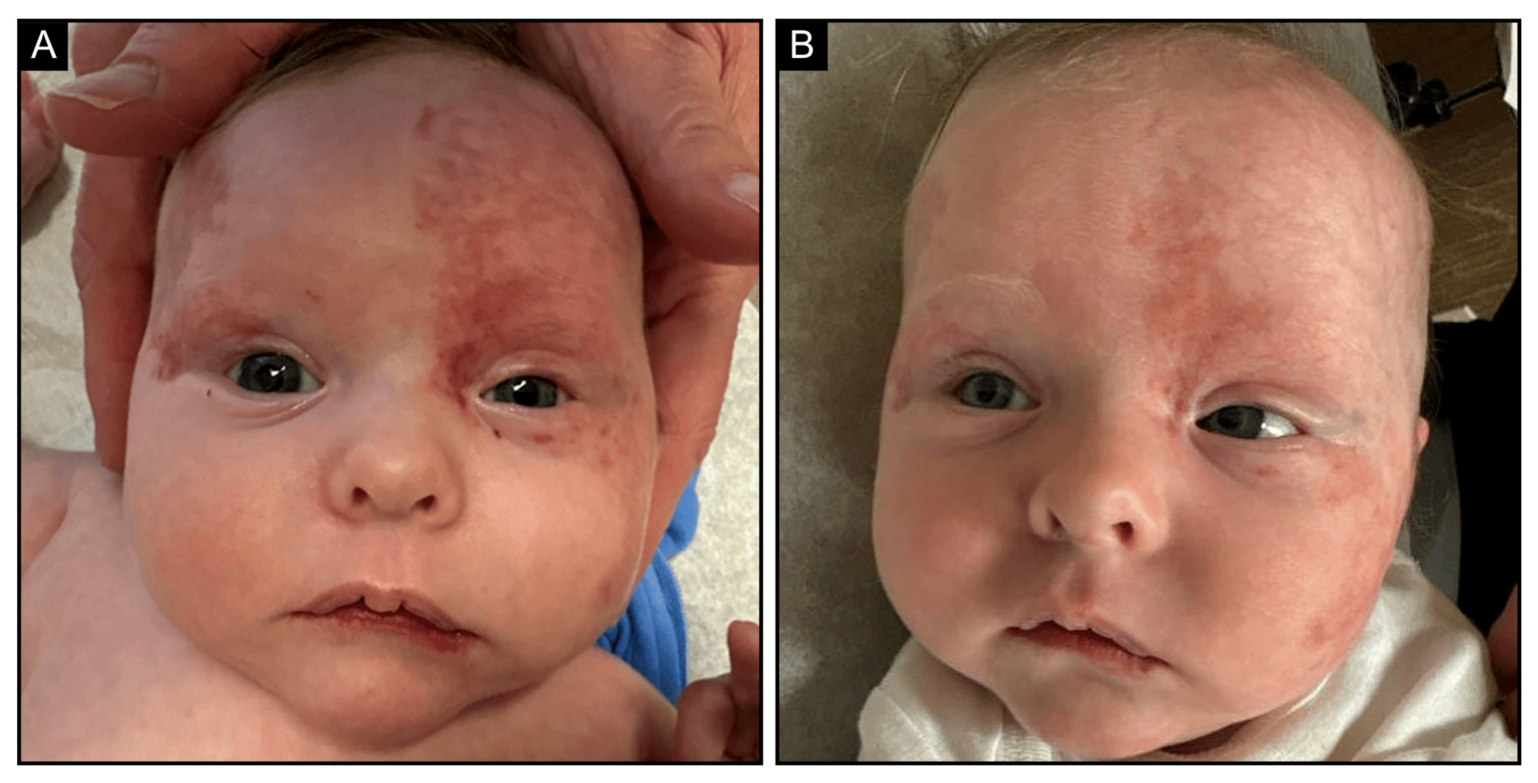
Infantile hemangioma (A) two weeks and (B) seven weeks following initiation of propranolol and prednisolone, demonstrating significant interval reduction in periocular swelling and improved left eye opening

Over the next several weeks, serial dermatology and ophthalmology visits noted significant improvement in the facial and periocular hemangiomas (Figure 4). The patient’s ability to open the left eye improved, and the abdominal/chest ulceration also improved.

At five months of age, the patient showed ongoing clinical improvement, with a significant reduction in hemangiomas and chest/abdominal ulceration, improved left eye opening, and age-appropriate developmental milestones. She currently takes propranolol (3 mg/kg/day), low-dose aspirin, and famotidine, with regular follow-up by dermatology, neurology, and ophthalmology. The patient was enrolled in a PHACE syndrome registry for long-term monitoring.

## Discussion

PHACE syndrome is a rare neurocutaneous disorder characterized by large, segmental facial infantile hemangiomas (IHs) and a constellation of congenital anomalies, including abnormalities in the brain, aorta, medium-sized vessels in the chest, neck, and head [2]. Prevalence is estimated to be less than one per million children; however, among infants who present with IHs, the prevalence is 2%-3% [3]. Observational studies have shown that prevalence increases to 20%-31% if the IHs are large, segmental, and located on the face [4]. Table 1 summarizes this patient's findings mapped to the formal diagnostic criteria established by Garzon et al. [2].

**Table 1:** Patient findings mapped to PHACE syndrome diagnostic criteria The diagnostic criteria were adapted from Garzon et al. [2]. MCA: Middle cerebral artery; PCA: Posterior cerebral artery; ASD: Atrial septal defect; PHACE: Posterior fossa brain malformations, hemangiomas, arterial anomalies, cardiac defects, and eye abnormalities.

Diagnostic Component	Finding in This Patient	Criterion Level	Diagnostic Significance
Hemangioma (required)	Large segmental facial hemangioma with bilateral periocular involvement	Required	Prerequisite for diagnosis; lesion must be >5 cm in diameter of the head/scalp
Arterial anomalies	Tortuous extracranial carotid arteries, dysplastic left MCA and PCA, and high-flow vascular malformations	Major	Confers high-risk status for arterial ischemic stroke; prompted initiation of low-dose aspirin
Structural brain	Dandy-Walker malformation, white matter deficiency, and periventricular neuronal heterotopia	Major	Associated with risk of developmental delay, seizures, and hydrocephalus; warrants neurology follow-up
Cardiovascular	Moderate atrial septal defect and patent ductus arteriosus	Associated finding (not a formal criterion)	ASD is listed as a rare finding in PHACE; warrants cardiology monitoring
Ocular	Bilateral orbital hemangiomas (left > right)	Minor	Risk of amblyopia from visual axis obstruction; treated with timolol drops
Ventral/Midline	Supraumbilical raphe	Major	Classic midline defect associated with PHACE; initially misidentified as a birth laceration

Typically, the IHs associated with PHACE syndrome are greater than 5 cm and involve one or more facial segments, most commonly in the frontotemporal (S1) or mandibular (S3) segments [4,5]. One prospective study found that patients with facial IHs larger than 22 cm^2 ^had an overall 31% risk of being diagnosed with PHACE syndrome [6]. IHs can appear as telangiectasia, solitary lesions, confluent plaques, or small papules that may be absent or faintly present at the time of birth and proliferate over the first several months of life. The mean age at diagnosis is approximately three months [4]. This has practical implications, as patients are typically not diagnosed at birth, and workup for anomalies of the brain, aorta, and thoracic or cervical arteries may be delayed, which can carry a significant risk of morbidity [3]. The American Academy of Pediatrics Clinical Practice Guidelines recommend that infants presenting with large, segmental facial hemangiomas (greater than 5 cm) should be promptly evaluated for PHACE syndrome [7].

Although IHs are the hallmark finding, PHACE syndrome often involves a wide range of extracutaneous manifestations. Cerebrovascular anomalies are particularly important, with arterial dysgenesis, stenosis, or agenesis of the cerebral arteries predisposing patients to arterial ischemic stroke and long-term neurodevelopmental complications [3,8]. According to the Garzon et al. (2016) consensus criteria, the dysplastic segments of the left MCA and PCA identified in this patient, in the setting of multiple arterial abnormalities, placed her in the high-risk category for arterial ischemic stroke, directly informing the decision to initiate prophylactic low-dose aspirin at 4 mg/kg/day [2]. Structural brain malformations may also be present, including cerebral hypoplasia, Dandy-Walker malformation, abnormalities of the fourth ventricle or pituitary gland, and, less commonly, intracranial hemangiomas [8,9]. Cardiac involvement frequently centers on defects of the aortic arch and its branches, such as coarctation of the aorta or an aberrant origin of the subclavian artery [6,10,11]. Additional associations include ocular abnormalities, which are most often posterior segment defects or optic nerve hypoplasia, as well as ventral midline defects such as a sternal cleft or supraumbilical raphe [4,8].

While the pathogenesis of PHACE syndrome is unknown, it is thought to be a result of defective embryogenesis between weeks 3 and 12 of gestation, either before or during vasculogenesis [3,12]. This is because the location of certain malformations and the IHs can affect the same side of the body [3].

If PHACE syndrome is suspected, patients should undergo an echocardiogram to evaluate for cardiac abnormalities and, if the echocardiogram has abnormal findings, subsequent cardiac MRI and MRA should be performed to better evaluate the cardiovascular anatomy [1,11]. Additionally, a gadolinium MRI and MRA of the brain, neck, and aortic arch should be performed in cases of suspected PHACE syndrome [7]. There is no established consensus regarding the optimal frequency of imaging. As a result, it is generally determined on a case-by-case basis, with high-risk patients potentially undergoing imaging as frequently as every three months [1].

Early recognition of the syndrome and comprehensive evaluation are vital to optimal outcomes for these patients; primary care providers must be aware of the presenting signs and promptly refer to the appropriate specialists [1,7]. Propranolol is the first-line medication for IH, and its use in patients with PHACE syndrome is based on the presence of extracutaneous anomalies and recommendations from their multidisciplinary team [7,13]. In this patient, propranolol was initiated at 0.5 mg/kg/day with inpatient monitoring in the PICU and slowly uptitrated to 3 mg/kg/day, consistent with the recommendations of Garzon et al. (2016) for high-risk patients, which emphasize a gradual dose escalation schedule to minimize sudden hemodynamic changes [2]. There is controversy around the use of propranolol in patients with severe arterial disease due to the risk of adverse effects such as hypotension, bradycardia, and potential cardiovascular accident [1,13]. However, its use can be quite effective in inducing regression of the hemangioma and is considered in those who have a high risk of ulceration, impairment of vital functions such as vision or airway compromise, or permanent disfiguration [1].It is typically started in the outpatient setting; however, inpatient initiation was elected in this case, given her high-risk cerebrovascular findings and elevated stroke risk. The initial dose is 0.5-1 mg/kg/day for one week and is increased to 2-3 mg/kg/day twice or three times daily [3]. Long-term outcomes are poorly described for patients with PHACE syndrome; however, prompt recognition and multidisciplinary assessment to identify CNS, cardiac, ocular, auditory, or developmental abnormalities in a timely manner can allow for effective interventions to prevent further complications [3,14].

## Conclusions

Our case illustrates the diverse and multisystem manifestations of PHACE syndrome. It underscores the importance of early recognition of large or ulcerated facial hemangiomas as potential indicators of PHACE syndrome. Timely diagnosis enables appropriate imaging, multidisciplinary involvement, and early initiation of targeted therapies, resulting in improved clinical outcomes. Our patient’s favorable response to treatment emphasizes the importance of early diagnosis and intervention in reducing complications such as amblyopia, ulceration, and stroke risk. This case also reinforces the need for primary providers to remain vigilant for cutaneous signs of systemic disease and to coordinate appropriate referrals to optimize outcomes. Increased awareness among providers may support earlier intervention and improved outcomes for affected infants.
